# Highly Diastereoselective Synthesis of Spiropyrazolones

**DOI:** 10.3390/molecules20058574

**Published:** 2015-05-13

**Authors:** Victor Ceban, Temitope O. Olomola, Marta Meazza, Ramon Rios

**Affiliations:** 1 Faculty of Natural & Environmental Sciences, University of Southampton Highfield Campus, Southampton SO17 1BJ, UK; E-Mails: vc2g12@soton.ac.uk (V.C.); tolomola@gmail.com (T.O.O.); mm3v12@soton.ac.uk (M.M.); 2 Department of Chemistry, Obafemi Awolowo University, Ile-Ife 220005, Nigeria

**Keywords:** spiropyrazolones, diastereoselective, cascade reaction

## Abstract

We report a highly diastereoselective synthesis of spiropyrazolones catalyzed by secondary amines. The reported Michael-Aldol cascade reaction affords the desired spiropyrazolones bearing four chiral centers as a single diastereomer in excellent yields.

## 1. Introduction

**Figure 1:**
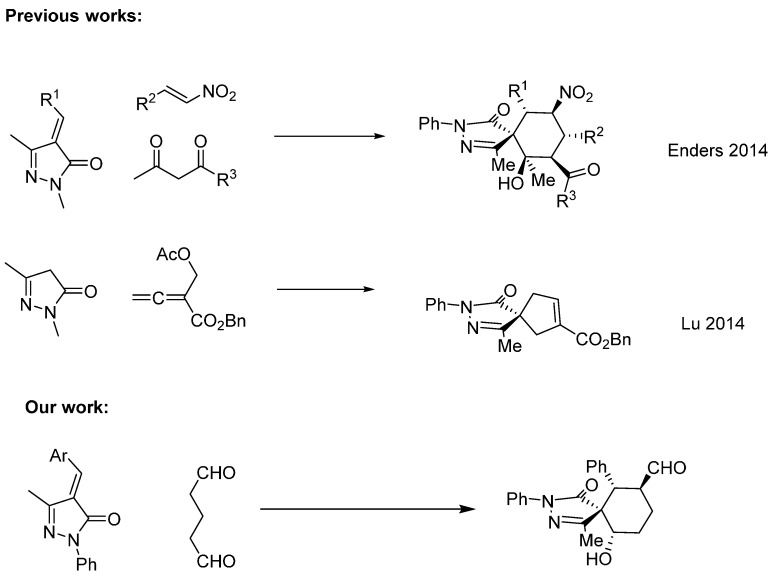
Previous reactions and our work.

The synthesis of spiro compounds [1] (formally bicyclic organic compounds with rings connected through just one atom) is one of the most important challenges that organic chemists are facing nowadays. Spiro compounds are highly important, not only because of their unique structural features in medicinal and natural products, but also for their use as catalysts in organic reactions with great success [2]. The first synthesis of spiro compounds can be traced back to seminal works of von Baeyer in XIX century. Since then, several chemists have developed efficient methodologies for their synthesis based on organometallic or organocatalytic approaches. In recent years, with the advent of organocatalysis, several highly diastereo- and enantioselective methodologies have been developed for their synthesis under the auspices of amino catalysis. For example, in 2009 Melchiorre developed a three component cascade Michael-Michael-aldol reaction between unsaturated oxindoles [3], enolizable aldehydes and enals catalyzed by Jørgensen-Hayashi catalyst; rendering the final spiro compound in excellent yields and stereoselectivities. One year later, in our research group we have developed a different Michael-Michael-aldol sequence leading to spiro compounds [4]. This time several heterocycles can be used (oxindoles, oxazolones, pyrazolones, *etc.*) in reaction with enals, achieving the final spiro compounds in excellent yields and in most of the examples with total stereoselectivity. Since then several research groups have been working designing new methodologies to afford highly substituted spirooxindoles [5,6,7]. Despite the growing interest in the synthesis of spirooxindoles, other heterocycles have started to blossom in this area due to their importance in pharmaceutical or agrochemical applications. Since the pioneering works of our research group in the highly stereocontrolled synthesis of pyrazolones via a Michael-Michael aldol reaction, several other groups developed similar strategies for the organocatalytic synthesis of spiropyrazolones with excellent results. For example, Lu and coworkers reported an elegant methodology for the synthesis of spiropyrazolones via a [4+1] cycloaddition catalyzed by chiral phosphines [8]; almost at the same time Enders showed the power of these approaches by developing a synthesis of spiropyrazolones with six vicinal centers in excellent yields and enantioselectivities [9]. Spurred by our interest in developing new asymmetric methodologies in the synthesis of spiro compounds [10,11] and inspired by these previous works, we envisioned an easy entry to spiropyrazolones by an organocatalyzed Michael-Aldol cyclization between methylenepyrazolones and dialdehydes (Figure 1) [12].

## 2. Results and Discussion

As a model reaction we choose methylenepyrazolone **1a** and the dialdehyde **2** that will get access to six-membered ring fused spiropyrazolones. We tested several solvents, catalysts and temperatures as is summarized in Table 1.

**Table 1:** Conditions screening. ^a^ 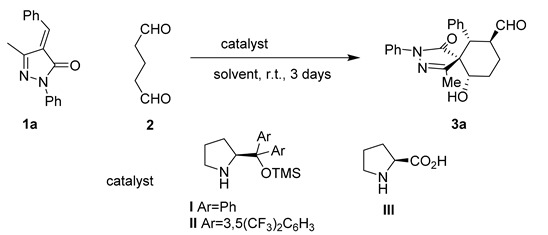

Entry	Solvent	Catalyst	Conversion ^b^	d.r. ^c^	e.r. ^d^
1	CH_2_Cl_2_	**I**	100%	7:1	66:34
2	Toluene	**I**	100%	10:1	66:34
3	*p-*Xylene	**I**	100%	10:1	53:47
4	AcOEt	**I**	100%	10:1	63:37
5	CHCl_3_	**I**	100%	5:1	53:47
6	THF	**I**	100%	5:1	52:48
7 ^e^	Toluene	**I**	100%	10:1	59:41
8	Toluene	**II**	n.r.	-	-
9	Toluene	**III**	n.r.	-	-
10 ^f^	Toluene	**I**	100%	2:1	66:34

^a^ For reaction conditions see general procedure; ^b^ Determined by ^1^H-NMR of the crude reaction; ^c^ Determined by ^1^H-NMR of the crude reaction; ^d^ Determined by HPLC analysis of the crude reaction; ^e^ 20 mol% of benzoic acid added; ^f^ Reaction carried out at −20 °C.

**Figure 2:**
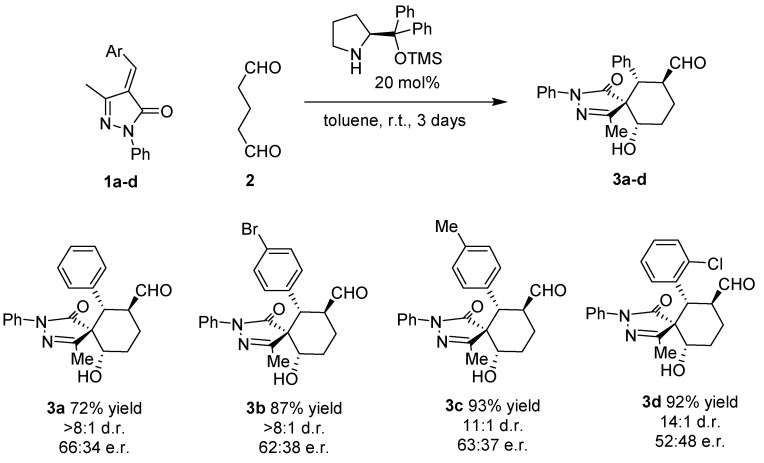
Reaction scope.

Despite the issues associated with the present reaction like autoaldolization of the dialdehyde, to our delight the reaction catalyzed by Jørgensen-Hayashi catalyst **I** rendered the final product in good conversion, good to excellent diastereoselectivities but low enantioselectivities in all the solvents tested. Better diastereoselectivities were obtained with aromatic solvents (toluene and *p*-xylene entries 2, 3 and 7; Table 1) achieving 10:1 d.r. but with low enantioselectivities (66:34). The use of other solvents such as CH_2_Cl_2_, CHCl_3_ or THF (entries 1, 5 and 6; Table 1) renders the final compounds with slightly worst diastereo- and enantioselectivities. The use of an additive such as benzoic acid have little effect on the outcome of the reaction only giving marginally worse enantioselectivities. Remarkably, the use of catalyst **II** did not render any cyclization product; when Proline (**III**) was used as catalyst the only product obtained was the aldol product of the dialdehyde (entries 8, and 9; Table 1). Decreasing the reaction temperature to −20 °C results in reduces the diastereoselectivity of the reaction without increasing the enantioselectivity. With the best conditions in hand we studied the scope of the reaction with different unsaturated pyrazolones. As it is shown in Figure 2, the reaction affords the final spiro compounds with excellent yields and diastereoselectivities but very low enantioselectivities.

As shown in Figure 2, the reaction works with several substituents in the para position Br (**3b**), Me (**3c**) rendering the final compound almost diastereopure in excellent yields. When a halogen was placed in the ortho position of the aromatic ring the diastereoselectivity was increased to 14:1 but we got an almost racemic compound (**3d**).

Next we tried the reaction using butandial as a dialdehyde source in order to have access to the cyclopentane derivatives. Unfortunately in all the reactions tested we only got decomposition of the starting material and no signals of any new product.

**Figure 3:**
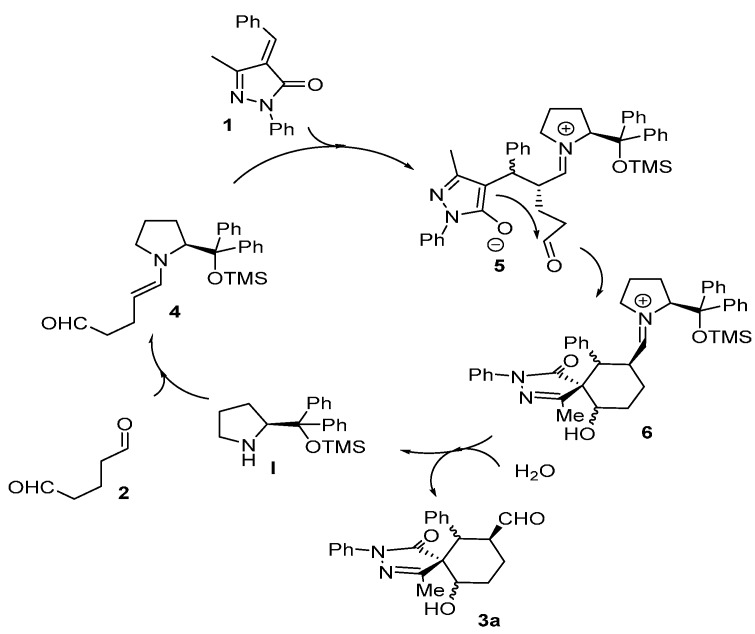
Proposed mechanism.

Surprisingly all the compounds in Figure 2 were obtained in extremely good diastereoselectivities but low enantioselectivities. This suggests an epimerization mechanism that renders the most stable diastereomer as product. The proposed mechanism for the reaction is shown in Figure 3.

**Figure 4:**
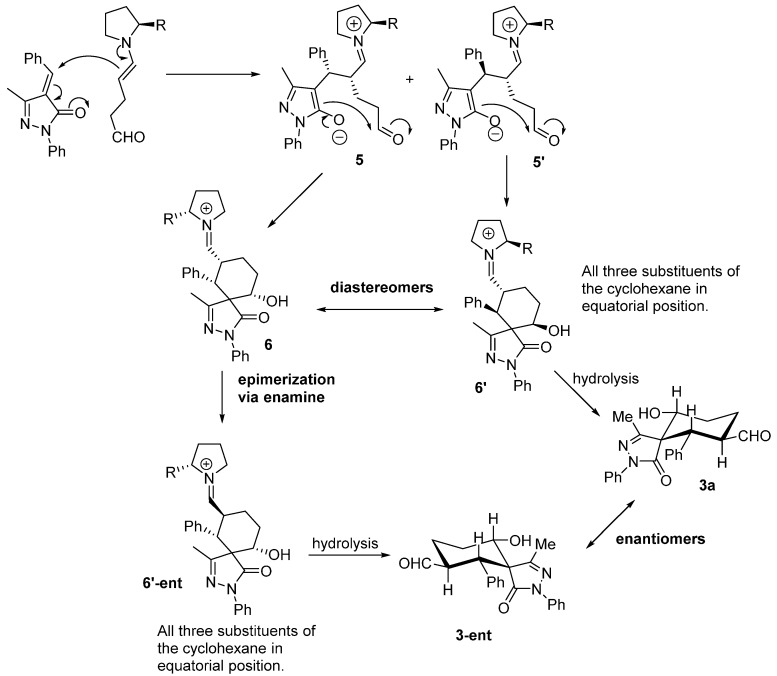
Plausible explanation for the low enantioselectivity observed.

The reaction starts with the enamine formation between dialdehyde **2** and the secondary amine catalyst (**I**). Next, the enamine **4** reacts with the Michael acceptor **1** to form adduct **5**. The catalyst efficiently shields one of the faces of the enamine, for this reason the attack takes place on the other side of the enamine. The formed enolate in the pyrazole ring undergoes an intramolecular aldol reaction with the other aldehyde group to form the intermediate **6**, which on hydrolysis renders the final product **3a** and regenerate the catalyst ready for a new catalytic cycle. Based on this mechanism what is expected is to have high enantioselectivities and poor diastereoselectivities. One possible explanation for the results obtained (poor ee and extremely good d.r.) could be the epimerization in the α-carbaldehyde position via an enamine intermediate. As shown in Figure 4 the first enamine addition should be highly stereospecific in the alpha position of the enamine because it is directly controlled by the catalyst, but probably we will get a mixture of diastereomers in the benzylic position (**5**, **5'**). The following aldol reaction will be termodynamically controlled affording the most stable compound in which the benzylic and the hydroxyl group present a relative equatorial position in the cyclohexane ring (**6**, **6'**). Next, an epimerization via enamine takes place to obtain the carbaldehyde substituent in a relative *trans* configuration with respect to the Ph in order to get all the substituents in relative equatorial positions of the cyclohexane ring. As is well known, Michael and aldol reactions are reversible reactions that will favour the most thermodynamically stable final compound.

**Figure 5:**
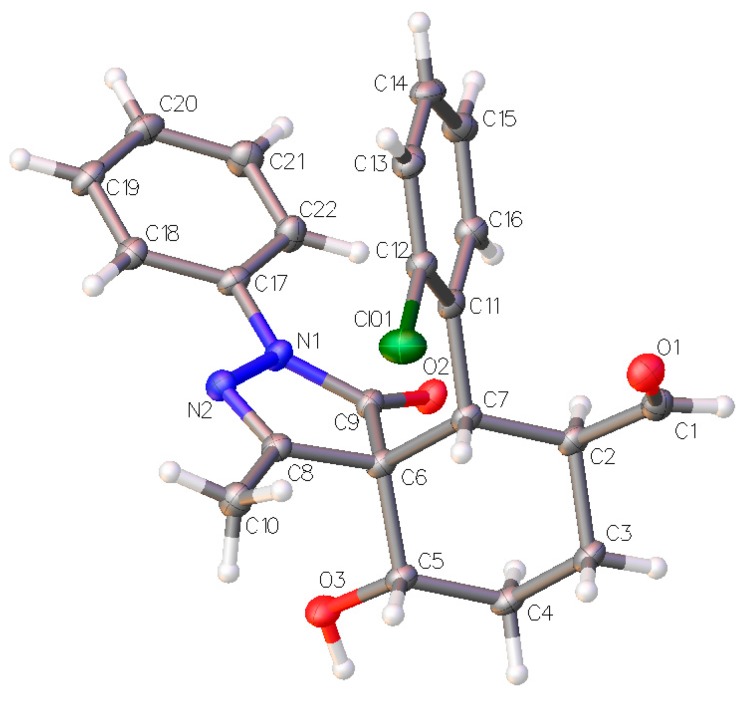
X-ray structure of compound **3d** (major diastereomer). The displacement ellipsoids are drawn at the 50% probability level [10].

The relative configuration of the major diastereomer was inferred by X-ray crystallography of compound **3d** (Figure 5) and confirms the proposed structure of compound **3a** where all the substituents are in equatorial position.

## 3. Experimental Section

General procedure for the preparation spyropyrazolones 3: To a solution of unsaturated pyrazolone (1) (1 equiv.) in toluene was added glutaraldehyde (3 equiv.) and (*S*)-2-(diphenyl ((trimethylsilyl) oxy) methyl)pyrroline (0.2 equiv.). The solution was stirred for 3 days and subsequently the solvent was removed by rotary evaporation. The reaction mixture was purified by column chromatography (Hexane/AcOEt). The product was confirmed by ^1^H-NMR.

**3a**: Purified by column chromatography (Hexane/AcOEt, 5:1).^1^H-NMR (CDCl_3_, 300 MHz): 9.59 ppm (d, 1H, ^3^*J* = 2.26 Hz), 7.78 ppm (dd, 2H, *J* = 1.13 Hz, *J* = 8.67 Hz), 7.57–7.45 ppm (m, 3H), 7.40–7.27 ppm (m, 5H), 4.12 ppm (ddd, 2H, ^3^*J* = 2.26 Hz, ^3^*J* = 3.39 Hz, ^3^*J* = 12.06 Hz), 3.27 ppm (d, 1H, ^3^*J* = 12.06 Hz), 2.83 ppm (ddd, 1H, *J* = 4.14 Hz, *J* = 13.19 Hz, *J* = 26 Hz), 2.34 ppm (s, 3H), 2.20–2.10 ppm (m, 2H), 1.80–163 ppm (m, 2H). ^13^C-NMR (CDCl_3_, 75 MHz): 203.0, 172.6, 160.5, 137.4, 135.8, 128.8, 128.7, 127.9, 125.4, 119.7, 71.9, 64.0, 47.5, 46.7, 27.0, 24.5, 13.6. HRMS (ESI) calcd. for C_22_H_23_N_2_O_3_ (M+H)^+^ 363.1703, found 363.1710. The enantiomeric excess was determined by HPLC using a Chiralpak ID column (hexane/*i*PrOH = 80:20, flow rate 1.0 mL/min, λ = 210 nm): t_r1_ = 13.4, t_r2_ = 15.6, 32% *ee.*

**3b**: Purified by column chromatography (Hexane/AcOEt, 3:2).^1^H-NMR (CDCl_3_, 300 MHz): 9.25 ppm (d, 1H, ^3^*J* = 1.88 Hz), 7.48 ppm (d, 2H, *J* = 7.91 Hz), 7.28–7.10 ppm (m, 5H), 6.92 ppm (d, 2H, *J* = 8.67 Hz), 3.75 ppm (ddd, 2H, ^3^*J* = 1.9 Hz, ^3^*J* = 3.8 Hz, ^3^*J* = 12. Hz), 2.92 ppm (d, 1H, ^3^*J* = 11.7 Hz), 2.44 ppm (ddd, 1H, ^3^*J* = 4.1 Hz, ^3^*J* = 13. Hz, ^3^*J* = 26.0 Hz), 1.97 (s, 3H), 1.83–1.73 ppm (m, 2H), 1.3 ppm (d, 2H, *J* = 3.77 Hz, *J* = 13.19 Hz, *J* = 22.23 Hz). ^13^C-NMR (CDCl_3_, 75 MHz): 201.3, 171.4, 159.3, 136.2, 134.1, 130.8, 127.7, 124.4, 121.0, 118.5, 70.6, 62.7, 45.7, 45.4, 25.7, 23.3, 12.4. HRMS (ESI) calcd. for C_22_H_22_Br^79^N_2_O_3_ (M+H)^+^ 441.0808, found 441.0812. The enantiomeric excess was determined by HPLC using a Chiralpak ID column (hexane/*i*PrOH = 80:20, flow rate 1.0 mL/min, λ = 210 nm): t_r1_ = 11.5, t_r2_ = 12.7, 24% *ee*.

**3c**: ^1^H-NMR (400 MHz, CDCl_3_) δ 9.38 (d, *J* = 2.0 Hz, 1H), 7.66–7.60 (m, 2H), 7.37–7.30 (m, 2H), 7.15 (t, *J* = 7.4 Hz, 1H), 7.06 (d, *J* = 8.0 Hz, 2H), 6.96 (d, *J* = 8.0 Hz, 2H), 3.94 (dd, *J* = 11.6, 4.6 Hz, 1H), 3.88 (tdd, *J* = 12.0, 3.6, 2.0 Hz, 1H), 3.02 (d, *J* = 12.0 Hz, 1H), 2.61 (ddd, *J* = 25.2, 13.3, 4.1 Hz, 1H), 2.22–2.16 (overlapping signals, 4H), 2.13 (s, 3H), 1.97–1.88 (m, 1H) and 1.49 (ddd, *J* = 26.1, 13.3, 4.1 Hz, 1H). ^13^C-NMR (100 MHz, CDCl_3_) δ 203.2, 172.7, 160.5, 137.8, 137.4, 132.6, 129.5, 128.6, 127.7, 125.3, 119.6, 71.9, 64.1, 47.2, 46.8, 26.9, 24.4, 20.9, 13.5. HRMS (ESI)calcd. for C_23_H_25_N_2_O_3_ (M+H)^+^ 377.1860, found 377.1856. The enantiomeric excess was determined by HPLC using a Chiralpak ID column (hexane/*i*PrOH = 80:20, flow rate 1.0 mL/min, λ = 210 nm): t_r1_= 12.1, t_r2_ = 14.3, 26% *ee.*

**3d**: ^1^H-NMR (400 MHz, CDCl_3_) δ 9.30 (d, *J* = 2.2 Hz, 1H), 7.71 (dd, *J* = 7.4, 6.3 Hz, 2H), 7.42–7.30 (m, 4H), 7.18 (t, *J* = 7.4 Hz, 1H), 7.08 (td, *J* = 7.6, 1.5 Hz, 1H), 7.02 (td, *J* = 7.6, 1.5 Hz, 1H), 4.06–3.98 (m, 1H), 3.88 (d, *J* = 11.9 Hz, 1H), 3.76 (tdd, *J* = 11.9, 3.4, 2.4 Hz, 1H), 2.65 (ddd, *J* = 25.4, 13.3, 4.3 Hz, 1H), 2.19 (s, 3H), 2.17–2.13 (m, 1H), 2.11 (d, *J* = 6.1 Hz, 1H), 1.95 (ddd, *J* = 9.2, 7.5, 4.1 Hz, 1H) and 1.59 (ddd, *J* = 25.4, 13.3, 4.1 Hz, 1H).^13^C-NMR (100 MHz, CDCl_3_) δ 202.1, 172.8, 161.0, 137.4, 134.0, 133.5, 130.0, 129.2, 128.8, 128.5, 127.6, 125.4, 119.5, 72.2, 63.3, 48.0, 41.9, 26.7, 24.4, 13.7. HRMS (ESI) calcd. for C_22_H_22_Cl_35_N_2_O_3_ (M+H)^+^ 397.1313, found 397.1308. The enantiomeric excess was determined by HPLC using a Chiralpak ID column (hexane/*i*PrOH = 80:20, flow rate 1.0 mL/min, λ = 210 nm): t_r1_ = 14.1, t_r2_ = 16.4, 3% *ee*.

X-ray: Single clear colourless fragment-shaped crystals of **3d** were recrystallised from a mixture of TCM and hexane by slow evaporation. A suitable crystal (0.09 × 0.08 × 0.05 mm^3^) was selected and mounted on a MITIGEN holder in perfluoroether oil on a Rigaku AFC12 FRE-HF diffractometer (University of Southampton, Southampton, UK). The crystal was kept at T = 100(2) K during data collection. Using Olex, the structure was solved with the ShelXT structure solution program, using the Direct Methods solution method. The model was refined with version of ShelXL using Least Squares. Unit cell parameters were refined against all data. An empirical absorption correction was carried out using CrystalClear software (University of Southampton, Southampton, UK). The crystal structure of **3d** was solved by charge flipping methods and refined on Fo^2^ by full-matrix least-squares refinements using programs of the SHELX-2013 software (University of Southampton, Southampton, UK). All non-hydrogen atoms were refined with anisotropic displacement parameters. All hydrogen atoms were added at calculated positions and refined using a riding model with isotropic displacement parameters based on the equivalent isotropic displacement parameter (U_eq_) of the parent atom. Single clear colourless fragment-shaped crystals of were recrystallised from a mixture of TCM and hexane by slow evaporation.

Crystal Data for C_22_H_21_ClN_2_O_3_ (*M* = 396.86 g/mol): monoclinic, space group P2_1_/n (no. 14), a = 9.90175(13) Å, b = 8.02985(9) Å, c = 23.3685(3) Å, β = 96.4448(12), *V* = 1846.28(4) Å^3^, *Z* = 4, T = 100(2) K, μ(CuKα) = 2.056 mm^−1^, *Dcalc* = 1.428 g/cm^3^, 24601 reflections measured (9.36° ≤ 2Θ ≤ 138.082°), 3363 unique (*R*_int_ = 0.0868, R_sigma_ = 0.0313) which were used in all calculations. The final *R*_1_ was 0.0456 (I > 2σ(I)) and *wR*_2_ was 0.1231 Crystallographic data (excluding structure factors) for the structure **3d** have been deposited with the Cambridge Crystallographic Data Centre with CCDC number 1062126. Copies of the data can be obtained, free of charge, on application to Cambridge Crystallographic Data Centre, 12 Union Road, Cambridge CB2 1EZ, UK, (fax: +44-(0)1223-336033 or e-mail: deposit@ccdc.cam.ac.uk).

## 4. Conclusions

In summary, we developed a new organocascade reaction for the synthesis of spiropyrazolones via a Michael-Aldol recation. The final spiropyrazolones were obtained in excellent yields and diastereoselectivities but with low enantioselectivities. The mechanistic studies, synthetic applications, and development of new organocascade reactions based on this concept are currently ongoing in our laboratory.
